# Efficacy of smallpox vaccines against Mpox infections in humans

**DOI:** 10.1093/immadv/ltad020

**Published:** 2023-10-07

**Authors:** Melissa M Christodoulidou, Neil A Mabbott

**Affiliations:** Edinburgh Medical School, Department of Biomedical Sciences, University of Edinburgh, Edinburgh, UK; The Roslin Institute & Royal (Dick) School of Veterinary Studies, University of Edinburgh, Easter Bush, Midlothian, UK

**Keywords:** Mpox, efficacy, vaccines, infections, humans

## Abstract

The Mpox virus (MPXV) is endemic in certain countries in Central and West Africa, where several mammalian species, especially rodents, are natural reservoirs. However, the MPXV can infect nonhuman primates and cause zoonotic infections in humans after close contact with an infected animal. Human-to-human transmission of MPXV can also occur through direct close contact with an infected individual or infected materials. In May 2022 an initial cluster of human Mpox cases was identified in the UK, with the first case confirmed in a patient who had recently travelled to Nigeria. The infection subsequently spread via human-to-human transmission within the UK and Mpox cases began to appear in many other countries around the world where the MPXV is not endemic. No specific treatments for MPXV infection in humans are available. However, data from studies undertaken in Zaire in the 1980s revealed that those with a history of smallpox vaccination during the global smallpox eradication campaign also had good cross-protection against MPXV infection. However, the vaccines used during the global eradication campaign are no longer available. During the 2022 global Mpox outbreak over a million doses of the Modified Vaccinia Ankara–Bavarian Nordic (MVA–BN) smallpox vaccine were offered either as pre or postexposure prophylaxis to those at high risk of MPXV infection. Here, we review what has been learned about the efficacy of smallpox vaccines in reducing the incidence of MPXV infections in high-risk close contacts.

## Introduction

The Mpox virus (MPXV; previously known as monkeypox virus) is a double-stranded DNA virus of the *Orthopoxvirus* genus of the *Poxviridae* family. The virus is endemic in certain Central and West African countries, where various mammalian species especially rodents, squirrels, prairie dogs, and rabbits serve as natural reservoirs. However, the MPXV can also infect nonhuman primates, and as the virus is zoonotic, spillover infections can be established in humans after close contact with an infected animal such as via bites, scratches, and handling infected tissues. Human-to-human transmission of MPXV can also occur through direct close contact with an infected individual, for example via broken skin lesions, mucous membranes, large respiratory droplets, body fluids, or contact with infected clothing or bedding.

In May 2022 an initial cluster of human Mpox cases was identified in London, UK, with the first case confirmed in a patient who had recently travelled to Nigeria [1]. Soon afterwards further Mpox cases were identified in individuals without a history of travel to regions where the MPXV is endemic. This suggested that human-to-human transmission of the MPXV had been established, and human Mpox cases subsequently began to be reported in many other countries around the world. As a consequence, on 23 July 2022, the World Health Organization (WHO) declared the global Mpox outbreak to be a public health emergency of international concern following reports of outbreaks in multiple countries where the MPXV is not endemic [2]. The majority of the cases in the global 2022 outbreak were detected amongst men who identified as gay, bisexual, or have sex with men, with transmission likely to have occurred via direct close contact during sexual activity.

Illness following MPXV infection is usually self-limiting in otherwise healthy individuals and typically lasts from two to four weeks [3]. The first clinical signs of MPXV infection in humans develop within 5 to 21 days of infection and include a fever, headache, muscle aches, swollen lymph nodes, and fatigue. Within a few days after the onset of fever, infected individuals can then develop a rash, often starting on or around the face, and can spread to other parts of the body including the groin. Individuals are infectious from the time that the rash starts to develop, but some are infectious from the onset of fever. The rash then develops into the characteristic pox blisters. People are no longer infectious once the blisters heal and the scabs slough off.

**Figure 1.**
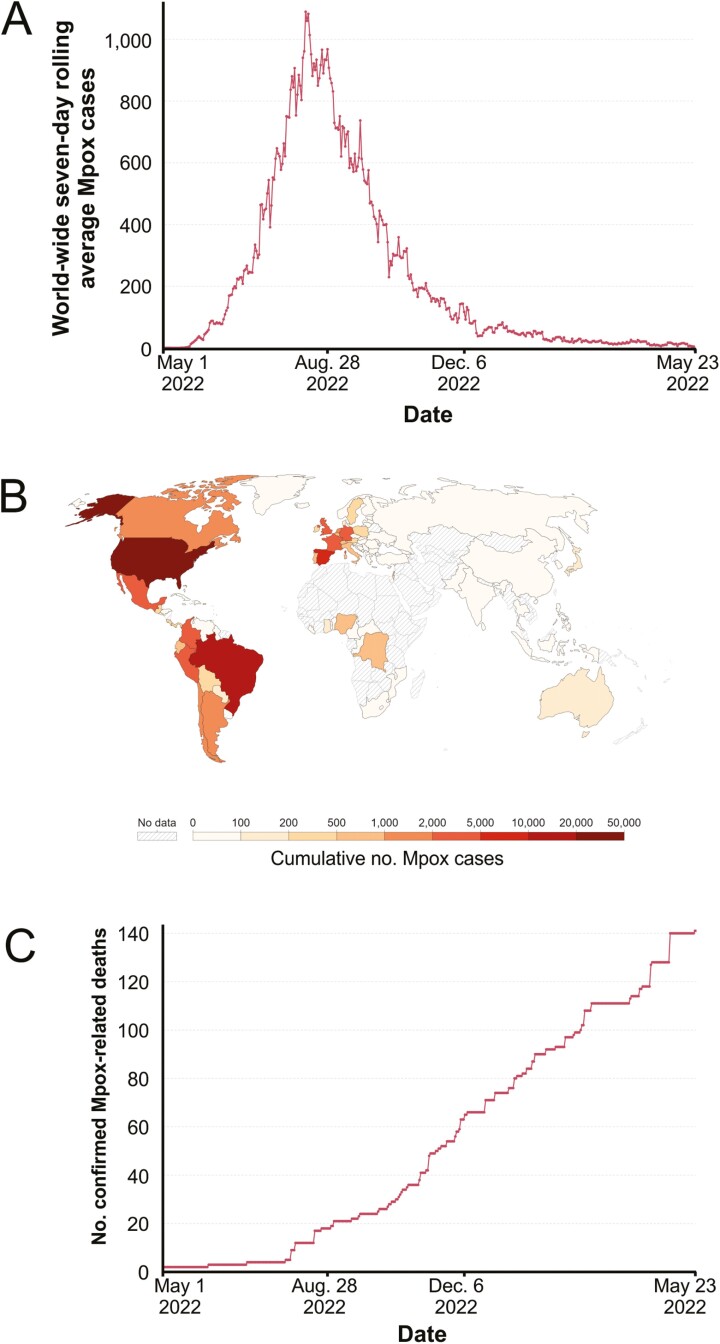
Confirmed human Mpox cases and deaths during the 2022 global outbreak. (**A**) Seven-day rolling average number of confirmed Mpox cases. (**B**) Geographical distribution of Mpox cases. (**C**) Cumulative number of confirmed Mpox-related deaths world-wide. Data reported by the World Health Organization between 1 May 2022 and 23 May 2023. Data retrieved from OurWorldInData.org [5] and adapted under the terms of the Creative Commons CC BY 4.0 Licence; https://creativecommons.org/licenses/by/4.0/).

Three MPXV clades have been identified [4]. Clade I is endemic to the Congo basin of Africa and has a case fatality rate in humans of up to 10%. Clade IIa, in contrast, is endemic in West Africa and is associated with a much lower-case fatality rate of <1%. Virus genome sequencing data have classified the Mpox viruses in circulation during the 2022 global outbreak as Clade IIb. Although 87 529 Mpox cases had been confirmed across 110 countries at the time of writing (May 2023), fortunately only 141 Mpox-related deaths had been reported, the majority in immunocompromised individuals or those with other serious underlying conditions (Fig. 1; https://ourworldindata.org/monkeypox). These data, alongside results from experimental transmissions to wild-derived castaneous (CAST/EiJ) mice [6] indicate that the Clade IIb Mpox viruses have a much lower-case fatality rate in humans than the other virus clades.

The smallpox virus also belongs to the *Orthopoxvirus* genus and causes a serious and highly infectious disease in humans. The smallpox virus was considered eradicated in 1980 as a consequence of a global vaccination campaign that used vaccines based on the closely related vaccinia virus. No specific treatments for MPXV infection in humans are available. However, data from Zaire in the 1980s revealed that those who received a smallpox vaccine during the eradication campaign also had good cross-protection against MPXV infection [7, 8]. However, the majority of individuals who are under 50 years old today do not have a history of smallpox vaccination, and the vaccines originally used during the global eradication campaign are no longer available. During the 2022 global Mpox outbreak over 1 million doses of updated smallpox vaccines were offered as either pre and postexposure prophylaxis to groups of individuals considered at high risk of MPXV [9]. Here we briefly review what has been learned about their efficacy in reducing the incidence of MPXV infections in high-risk close contacts.

## Vaccination against smallpox during the global eradication campaign provided cross-protection against MPXV infection

Original data on the efficacy of smallpox vaccines against MPXV infection in humans derive from two separate studies of human Mpox cases in Zaire in the 1980s [7, 8]. Secondary MPXV infection rates were compared in unvaccinated close contacts and those who had a previous history of smallpox vaccination as part of the global eradication campaign.

In the first study, 1573 contacts of 147 primary Mpox patients (suspected to have been infected from an animal source) were analysed between 1980 and 1984 [6]. The overall secondary MPXV infection attack rate in individuals with a history of smallpox vaccination was 1.1%, compared to 7.4% in those who had not (Table 1). A separate study analysed 2278 close contacts of 245 Mpox patients between 1981 and 1986 [7]. The secondary infection attack rate amongst the unvaccinated close contacts was 7.47%, whereas the rate in those who were vaccinated was 0.96% (Table 1).

**Table 1.** Effects of previous smallpox vaccination on secondary MPXV infection attack rates in two studies of close contacts of primary Mpox cases in Zaire

Vaccination status of contacts	No. contacts^1^	No. secondary Mpox cases	Attack rate	Vaccine efficacy^4^	Reference
Study #1^2^					
Unvaccinated	474	35	7.4%		
Vaccinated	1099	12	1.1%	85.1%	[7]
Study #2^3^					
Unvaccinated	723	54	7.47%		
Vaccinated	1555	15	0.96%	87.1%	[8]

^1^Close contacts of primary Mpox patients suspected to have been infected from an animal source.

^2^Analysed 1573 contacts of 147 primary Mpox patients between 1980 and 1984 [6].

^3^Analysed 2278 close contacts of 245 Mpox patients between 1981 and 1986 [7].

^4^Estimated reduction in secondary Mpox cases in close contacts with a history of smallpox vaccination compared to unvaccinated close contacts.

These two studies provided similar estimates of the efficacy of the first-generation smallpox vaccines against MPXV infection. They suggested that previous vaccinations against smallpox during the global eradication campaign provided between 85.1% [7] and 87.1% [8] protection against Mpox in close, face-to-face contact with infected patients (Table 1). Prior smallpox vaccination also appeared to be similarly effective against primary MPXV infection. Only 12.7% of the primary Mpox patients analysed in the study by Jezek and colleagues had a history of smallpox vaccination [8].

## Second- and third-generation smallpox vaccines approved for use against Mpox infection

The individuals in the two studies described above were given first-generation smallpox vaccines which are no longer in use [7, 8]. Since then, updated smallpox vaccines have been designed with three given emergency approval for use against MPXV infection (Table 2).

**Table 2.** Licenced second and third-generation smallpox vaccines

Vaccine	ACAM2000	LC16m8	Modified vaccinia Ankara–Bavaria Nordic
Trade name			Jynneos (US)/ Imvamune (Canada)/ Imvanex (EU)
Manufacturer	Sanofi Pasteur Biologics	Kaketsuken	Bavarian Nordic
Vaccine type	Second generation	Third generation	Third generation
Virus type	Vaccinia strain	Lister strain of the vaccinia virus	Modified Vaccinia Ankara–Bavarian Nordic (MVA–BN) Strain
Replication status	Live attenuated, replication competent	Live attenuated, minimally replication competent	Live attenuated, non-replication competent
Approvals for use	Australia, Singapore, USA	Japan	Europe, Canada, USA

### ACAM2000 smallpox vaccine

Prior to the 2022 Mpox outbreak, the second-generation smallpox vaccine ACAM2000 (Emergent Biosolutions; https://www.emergentbiosolutions.com/wp-content/uploads/2022/01/ACAM2000-Product-Information.pdf/ Sanofi Pasteur) was the predominant formulation that was being retained in the USA for emergency use against smallpox [10]. However, this vaccine carries a risk of myocarditis and pericarditis in some recipients, and is unsuitable for use in those with weakened immune systems, very young, pregnant people, and those with certain skin conditions [10]. This is because the vaccine comprises a live vaccinia virus that can replicate and cause serious adverse reactions in these individuals. The efficacy of the ACAM2000 vaccine against MPXV infection is currently unknown. Fortunately, two-third-generation smallpox vaccines have been developed which have demonstrated improved safety profiles in clinical trials [11].

### LC16m8 smallpox vaccine

LC16m8 (Kaketsuken [12]) is a third-generation smallpox vaccine that uses a live, highly attenuated, replicating version of the Lister strain of the vaccinia virus. This vaccine was first used in Japan in the 1970s for routine vaccination against smallpox, with tens of thousands of vaccine doses distributed between 1974 and 1975, and no serious adverse events reported [12]. The vaccine achieves immunogenicity after a single dose, and two experimental studies show it can protect against lethal MPXV infection in nonhuman primates [13, 14]. How effective LC16m8 is against MPXV infection in humans is uncertain. We may eventually gain some insight from the 2022 Mpox outbreak as this vaccine has been offered as postexposure prophylaxis in some regions of Japan [15].

### Modified Vaccinia Ankara–Bavaria Nordic (MVA–BN) smallpox vaccine

**Figure 2.**
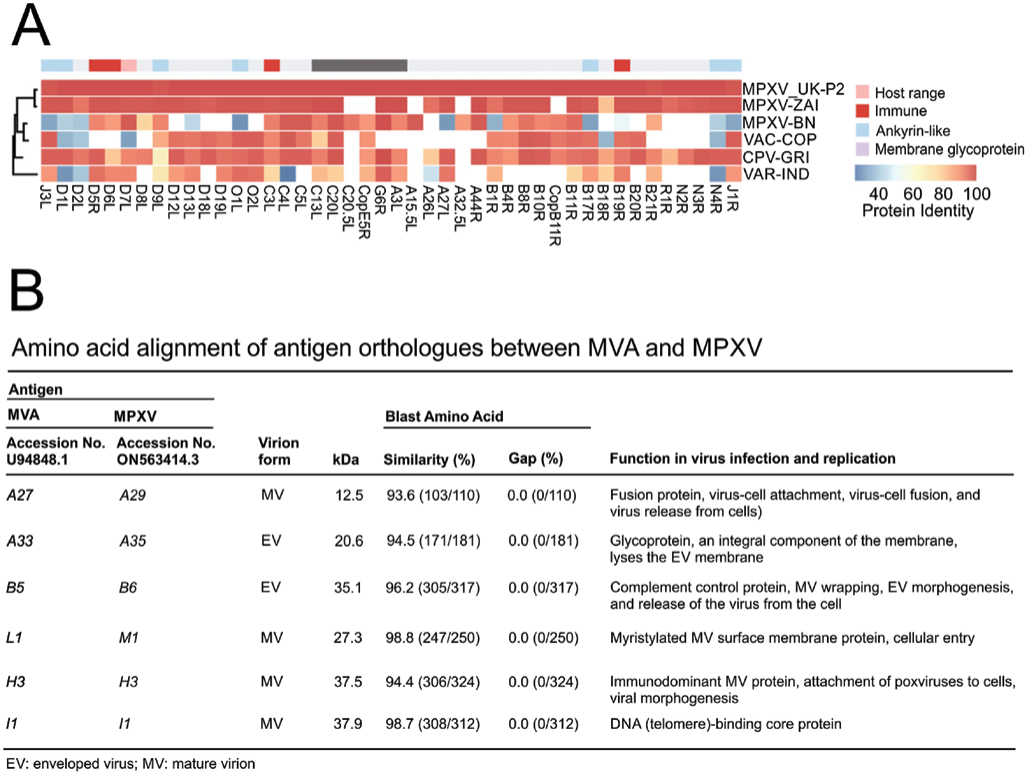
Protein sequence homology between the MVA–BN virus and MPXV-2022. (**A**) Homology between consensus protein sequences of MPXV-2022 (MPXV-UK-2), MPXV Zaire‐96‐I‐16 (MPV-ZAI), MVA–BN, vaccinia virus (VAC-COP), cowpox virus (CPV-GRI) and variola major virus strain India (VAR‐IND). *X* axis, virus proteins. (**B**) Comparison of amino acid sequence homology between orthologues of protective antigens of MVA (GenBank: U94848.1) and the current MPXV strain (GenBank: ON563414.3). Data in panel (**A)** are adapted from [16] and panel (**B)** are adapted from [17] under the terms of the Creative Commons CC BY 4.0 Licence; https://creativecommons.org/licenses/by/4.0/).

The MVA–BN smallpox vaccine (Jynneos/Imvanex/Imvamune; Bavarian; https://www.bavarian-nordic.com/pipeline/technology/mva-bn.aspx) uses a live, attenuated strain of the vaccinia virus that cannot replicate in human cells. The genomes of the MVA–BN virus used in this vaccine and the 2022 MPXV virus strain (MPXV-2022) share a high degree of homology [16] with the protective antigens A29, A35, B6, M1, H3, and I1 displaying ≥93% amino acid sequence identity [17] (Fig. 2). In mice, vaccination with MVA or with the A29 or M1 virus antigens induces significant neutralising antibody responses against the MPXV-2022 strain [17]. The MVA–BN vaccine has been approved for use against Mpox in many countries around the world including the UK, Europe, and in USA. Additional safety monitoring data has also been collected in the US during the 2022 Mpox outbreak (between 22 May 2022 and 21 October 2022) from recipients of almost 1 million vaccine doses [9]. The most common adverse reactions following vaccination were nonserious such as injection site reactions. Serious adverse events were rare, and none were identified in children under 18 years old [9]. Two 0.5 ml doses of the vaccine administered subcutaneously 4 weeks apart are recommended to achieve optimal immunity. Reducing the vaccine dose to 0.1 ml was not considered to affect the magnitude of the immune response [18]. As a consequence, some countries including the UK, the USA, and the EU recommended reducing the vaccine dose to 0.1 ml to extend supplies [19]. However, a subsequent study has suggested that two doses of MVA–BN given four weeks apart induce relatively low serum MPXV-neutralising antibody levels [20]. Whether the magnitude of the neutralizing antibody response induced after MVA–BN vaccination affects the protection against MPXV infection is uncertain.

## Efficacy of the MVA–BN smallpox vaccine as pre-exposure prophylaxis against Mpox infection

At the time of writing two studies had reported data on the efficacy of the MVA–BN smallpox vaccine when used as preexposure prophylaxis against MPXV infection in high-risk individuals [21, 22].

### Israel

Sagy and her colleagues evaluated the effectiveness of a single, subcutaneous dose of the MVA–BN vaccine against MPXV infection in 2054 high-risk members of Clait Health Services, Israel [21]. All the individuals included in the study were males, and 50% (1037) were vaccinated and monitored for symptomatic Mpox for between 90 and 147 days afterwards. During the study period, five Mpox cases were confirmed in vaccinated individuals and 16 in those who were not (determined by laboratory RT-PCR test for the MPXV). All of the cases that were detected in the vaccinated individuals occurred between 21 and 47 days later, suggesting they were breakthrough infections. A Cox proportional hazards regression model with time-dependent covariates was used to estimate the association between vaccination and Mpox while adjusting for sociodemographic and clinical risk factors. Data from this study suggested that vaccination of high-risk individuals with one subcutaneous dose of MVA–BN was associated with an 86% reduction (95% CI, 59–95%) in the risk of MPXV infection. However, a preprint (that had not undergone peer-review at the time of writing) had presented an alternative analysis. In it, the authors argued that when differences in the duration of exposure to the MPXV between the groups of vaccinated and unvaccinated individuals were taken into consideration [23], vaccine efficacy may be as much as 50% lower than the original estimates suggest [21].

### England

A case-coverage study from England also aimed to assess the effectiveness of a single dose of the MVA–BN vaccine against symptomatic MPXV infection in high-risk individuals [22]. Confirmed Mpox cases were identified by laboratory reports [24] with follow-up questionnaires used to gain details on vaccination status, symptoms, and timing of rash onset. Amongst 362 confirmed Mpox cases, the majority occurred in unvaccinated individuals, with 32 cases detected ≤13 days after vaccination and only eight cases detected ≥14 days afterwards. The case-coverage method, which compared vaccine coverage among cases to the coverage in the eligible population, was used to estimate the vaccine effectiveness. This estimated that a single dose of the MVA–BN vaccine was 78% (95% CI, 54–89) against symptomatic Mpox infection from ≥14 days after vaccination [22]. However, data in the study also indicated that the vaccine provided negligible protection within the first 13 days after vaccination.

## Efficacy of the MVA–BN smallpox vaccine as postexposure prophylaxis against Mpox infection

At the time of writing four studies from France, Spain, and the USA had also assessed whether vaccination is similarly effective when used as postexposure prophylaxis (PEP) soon after high-risk exposure [25–28].

### France

In this study, 276 individuals were given a single dose of the MVA–BN smallpox vaccine as PEP within a median time of 11 days (range 0–16 days) after high-risk MPXV exposure [25]. High-risk exposure was defined as direct skin-to-skin or mucosal contact with an infected person, indirect contact with contaminated surfaces or textiles, exposure to respiratory droplets, or combinations of these types of exposure. Twelve Mpox cases were confirmed amongst the 276 participants (4%), 10 of which occurred within five days of vaccination, and the other two were detected 22 days and 25 days afterwards. Infections in individuals with signs of MPXV infection were confirmed by PCR for the MPXV. However, due to the absence of unvaccinated controls in this study, it is difficult to make conclusions on whether vaccination soon after high-risk exposure was effective as PEP against MPXV infection. The clinical signs of Mpox typically occur within 5–21 days after exposure. The early onset of the majority of the Mpox cases (10/12) within a median duration of five days after vaccination suggests that some of these individuals may already have been exposed to Mpox before entering the study.

### Spain

A further study from Madrid, Spain, analysed the efficacy of a single subcutaneous dose of the MVA–BN vaccine when used as PEP in close contact with laboratory-confirmed Mpox cases [28]. Here, the close contacts were those who had had contact with body fluids or lesions, or unprotected exposure to contaminated fomites, or clinical samples from a Mpox case since the appearance of their first symptoms. The study was conducted between 16 May 2022 and 15 August 2022, and the close contacts were monitored for up to 49 days after vaccination. Amongst the 484 contacts included in this study, 230 were vaccinated, and the remaining 254 were not. A total of 57 of these contacts developed Mpox during the follow-up period: Eight occurred in vaccinated contacts, compared to 49 in the unvaccinated contacts. This implied that the unvaccinated close contacts of Mpox cases had an 8.9 times higher risk of MPXV infection (95% CI, 4.2–19.1) than those who were. The authors estimated that PEP vaccination with a single dose of MVA–BN reduced the risk of developing Mpox disease in close contacts of Mpox cases by 88.8% (95% CI, 76.0–94.7).

### USA

Two related studies from the USA have also reported data on vaccine efficacy when used as PEP in cohorts of high-risk men aged 18-49 years old. The first study analysed data from 5402 reported Mpox cases across 32 US jurisdictions between 31 July 2022 and 3 September 2022 [26]. An extended follow-up study assessed data from 9544 reported Mpox cases from 43 US jurisdictions between 31 July 2022 and 1 October 2022, and used an updated statistical model to improve the accuracy of the vaccine efficacy estimates [26]. In each of the studies the vaccinated individuals had received one or more doses of the MVA-BN vaccine, and Mpox cases were confirmed by PCR testing, next-generation sequencing, or cultivation of the MPXV from clinical specimens.

Amongst the 5402 reported Mpox cases analysed in the first study [27], 4606 were reported in unvaccinated individuals. In vaccine recipients, in contrast, 269 Mpox cases occurred in those in which disease onset was first detected ≤13 days after the first vaccine dose, with just 77 cases identified with disease onset ≥14 days afterwards. These data suggested that the Mpox incidence (expressed as cases per 100 000) in high-risk unvaccinated persons were 14.3 times higher (95% CI, 5.0–41.0) than in those who had received a single vaccine dose ≥14 days previously. This implied that PEP vaccination with MVA–BN reduced the risk of MPXV infection by ~93%.

The larger follow-up study of 9544 reported Mpox cases across 43 US jurisdictions reported 8320 cases in unvaccinated individuals, compared to 1224 in vaccinated persons [26]. Amongst the vaccine recipients, 614 Mpox cases were detected ≤13 days after the first vaccine dose, 392 were first detected ≥14 days after the first vaccine dose, and 48 cases with illness onset ≥14 days after receipt of the second dose. This suggested that the incidence of Mpox cases (cases per 100 000 population at risk) in the unvaccinated individuals was 7.4 times higher (95% CI, 6.0–9.1) than in those who had received just one vaccine dose ≥14 days earlier, and 9.6 times higher (95% CI, 6.9–13.2) than in those who had received a second dose ≥ 14 days earlier. Data from this extended study imply that a single vaccine dose given as PEP ≥ 14 days earlier reduced the risk of Mpox infection in high-risk individuals by 86.5% (95% CI, 74.4–89%), and increased modestly to 89.5% (95% CI, 85.5–92.4%) in those who had received a second dose.

This study also allowed the impact of the route of vaccination to be compared, since some individuals were given their vaccines subcutaneously, and some intradermally [26]. However, the relative numbers of Mpox cases in individuals vaccinated by either route were not significantly different when compared to the overall vaccinated population (*P* = 0.28 [26]). This is useful, since by November during the 2022 outbreak, 65% of vaccine clinics in the UK were administering the vaccine intradermally [22].

## Humoral and cellular immunity induced by first-generation smallpox vaccinates is long-lived

How long the cross-protection against Mpox infection is maintained after smallpox vaccination is uncertain. However, follow-up longitudinal studies in smallpox vaccine recipients have revealed that serum anti-vaccinia virus neutralising antibody responses may be stably maintained for decades, perhaps life-long, in some individuals [29–31]. The presence of MPXV-neutralising antibodies has also been detected in the serum of individuals born before 1974 with a history of smallpox vaccination [20].

The longevity of cellular immunity, in contrast, is less certain. Vaccinia virus-specific T-cell responses in a study of US vaccine recipients were suggested to steadily decline with a half-life ranging from 8 to 15 years. Despite this gradual waning, evidence of both CD4^+^ and CD8^+^ T cell memory could be detected in many individuals up to 75 years after the last vaccination, but at reduced levels compared to those detected in previous years [30]. In a separate study of Taiwanese vaccine recipients, vaccinia virus-specific T-cell memory responses were reported to persist for up to 30 years after vaccination, eventually declining to the levels detected in unvaccinated individuals by 40 years [32].

Together, these studies suggest that vaccinia virus-specific humoral and cellular immunity can persist for decades in some individuals after historic smallpox vaccination. Some of the individuals who were vaccinated decades ago against smallpox during the global eradication programme may also have significant cross-protection (neutralising antibody responses) against MPXV infection.

## Concluding remarks

Studies undertaken during the 2022 Mpox outbreak show that a single subcutaneous dose of the MVA–BN smallpox vaccine is associated with a significantly lower risk of MPXV infection when used in high-risk close contacts as either pre [21, 22] or postexposure prophylaxis [26–28] (Table 3). The efficacy of the MVA–BN smallpox vaccine against MPXV infection appears to be remarkably similar to that reported for the original smallpox vaccines used during the global smallpox eradication campaign (78–88.8% vs. 85–87% reduction in MPXV infections, respectively [7, 8];). However, the recent studies do have some limitations that might affect vaccine efficacy. For example, almost all the individuals in these studies were males, and most were aged between 18 and 49 years old [21, 22, 26–28]. Whether the vaccines are similarly effective in females, the young (<18 years old) and the elderly remains to be determined. Many of the participants were also HIV-positive, and some may have had a significant degree of immunosuppression.

**Table 3.** Summary of efficacy of MVA–BN vaccination against secondary MPXV infection in high-risk close contacts of Mpox cases

Study	Vaccination method	No. vaccine doses	Estimated vaccine efficacy^1^	Reference
Israel	Pre-exposure prophylaxis	1	86% (95% CI, 59–95%)	[21]
England	Pre-exposure prophylaxis	1	78% (95% CI, 54–89%)	[22]
Spain	Post-exposure prophylaxis	1	88.8% (95% CI, 76.0–94.7)	[28]
USA (32 jurisdictions)	Post-exposure prophylaxis	1	93%	[27]
USA (43 jurisdictions)	Post-exposure prophylaxis	1	86.5% (95% CI, 74.4–89%)	[26]
USA (43 jurisdictions)	Post-exposure prophylaxis	2	89.5% (95% CI, 85.5–92.4%)	[26]

^1^Estimated reduction in secondary MPXV infections in vaccinated high risk close-contacts of Mpox cases compared to unvaccinated close-contacts.

The first insights into the efficacy of the cross-protection from smallpox vaccination against human MPXV infection were derived from Zaire where the Mpox virus is endemic in certain wildlife species [7, 8]. In contrast, the MVA-BN vaccine was assessed in recipients in high-income countries in the global north [21, 22, 26–28]. As many as 2 billion people in low and middle-income countries may be infected with helminth parasites [33]. The immune response to helminth infections can suppress immunity to infections with other pathogens and affect responses to certain vaccines [34, 35]. Therefore, it is important that participants in endemic African nations are included in future vaccine trials to account for the potential influence that additional factors such as co-infection with helminth parasites might have [36]. Furthermore, although the number of deaths among individuals with MPXV infection during the 2022 outbreak has fortunately been low (predominantly occurred in immunocompromised patients), it was striking that 86.8% of the deaths in one USA report had occurred in Black persons [37].

Novel SARS-CoV-2 virus variants of concern arose during the COVID-19 pandemic with the ability to escape some of the immunity gained from the original vaccines or previous infection [38]. This stimulated the development of updated vaccines to help provide cross-protection to SARS-CoV2 virus variants of concern such as Omicron [39]. Whether similar mutations are occurring in the MPXV genome and whether vaccine modifications may be required to maintain immunity in the future is uncertain. A study from the Loire Valley in France has recorded breakthrough Mpox cases in nine individuals ~6 months after vaccination [40]. Whether this is a consequence of waning immunity or the relatively low levels of MPXV-specific neutralizing antibody responses induced after MVA–BN vaccination [20], or indicates the emergence of new MPXV variants with the ability to escape vaccine-induced immunity remains to be determined. Although vaccination is considered to be ~85% effective, a small number of breakthrough cases are to be expected and were observed in the pre-exposure prophylaxis studies. For example, Sagy and colleagues detected five breakthrough Mpox infections between 21 and 47 days after one vaccine dose [21].

While vaccination against MPXV infection has relied on the use of smallpox virus-specific vaccines, those that specifically target MPXV are in experimental development. These include DNA-based vaccines encoding several conserved MPXV epitopes [41] or recombinant vaccines containing MPXV structural proteins [42, 43]. Furthermore, the recombinant vaccines were shown to provide protection against Mpox in experimentally infected mice or macaques [42, 43].

Together, the available evidence indicates that MVA–BN vaccination was a safe and effective way to protect against symptomatic Mpox infection in high-risk close contacts. Despite this, estimating the precise impact that this targeted vaccination approach had on MPXV infections and transmission during the 2022 outbreak is difficult since they were used alongside other outbreak control measures, including the isolation of infected patients, as well as increased education, awareness, and surveillance amongst high-risk groups. However, in the countries where the MVA–BN vaccine was provided, Mpox cases in high-risk individuals have continued to rapidly decline. For example, whereas there was 3732 confirmed and highly probable Mpox cases in the UK between 6 May 2022 and 31 December 2022, only 20 had been reported in 2023 (up to 24 May [44];). Similarly, in the USA over 30 000 Mpox cases were recorded in 2022, compared to just 203 during the same period in 2023 [45].

The World Health Organization declared an end to the public health emergency of international concern in May 2023 [46], and the Mpox vaccination campaign was scheduled to end in the UK in July 2023 [47]. Whether those who were given smallpox vaccinations during this period will require a subsequent booster to maintain immunity against the MPXV during the remainder of the outbreak remains to be determined. The combined influence of the waning of immunity from smallpox vaccination and the increasing number of unvaccinated individuals following the end of the global smallpox eradication campaign is considered to have contributed to the increased number of spillover human MPXV infections detected in some African countries in recent years [48]. This raises the question of whether routine smallpox vaccination in African countries where the MPXV is endemic would be an appropriate use of resources to help prevent future Mpox spillover events and limit the opportunities for the virus to become established in humans.

## Data Availability

Data sharing is not applicable to this article as no new data were created or analysed in this study.

## Abbreviations

CI: confidence interval
MPXV: Mpox virus
MVA-BN: Modified Vaccinia Ankara–Bavarian Nordic smallpox vaccine
PEP: post-exposure prophylaxis

## References

[1] World Health Organization. Monkeypox – United Kingdom of Great Britain and Northern Ireland, 2022. https://www.who.int/emergencies/disease-outbreak-news/item/2022-DON381

[2] World Health Organization. WHO Director-General declares the ongoing monkeypox outbreak a Public Health Emergency of International Concern, 2022. https://www.who.int/europe/news/item/23-07-2022-who-director-general-declares-the-ongoing-monkeypox-outbreak-a-public-health-event-of-international-concern

[3] World Health Organization. Mpox (monkeypox), 2022. https://www.who.int/news-room/fact-sheets/detail/monkeypox

[4] World Health Organization. Monkeypox: experts give virus variants new names, 2022. https://www.who.int/news/item/12-08-2022-monkeypox--experts-give-virus-variants-new-names

[5] Mathieu E , Spooner F, Dattani S et al. Mpox (monkeypox) 2022. OurWorldInData.org https://ourworldindata.org/monkeypox

[6] Americo JL , Earl PL, Moss B. Virulence differences of mpox (monkeypox) virus clades I, IIa, and IIb1 in a small animal model. Proc Natl Acad Sci USA 2023;120(8):e2220415120. 10.1073/pnas.2220415120 36787354 PMC9974501

[7] Fine PEM , Jezek Z, Grab B et al. The transmission potential of monkeypox virus in human populations. Int J Epidemiol 1988;17(3):643–50. 10.1093/ije/17.3.643 2850277

[8] Jezek Z , Grab B, Szczeniowski MV et al. Human monkeypox: secondary attack rates. Bull World Health Organ 1988;66(4):465–70. 2844429 PMC2491159

[9] Duffey J , Marquez P, Moro P et al. Safety monitoring of JYNNEOS vaccine during the 2022 Mpox outbreak - United States, May 22 - October 21, 2022. Morb Mortal Wkly Rep 2022;71(49):1555–9. 10.15585/mmwr.mm7149a4 PMC9762898 36480476

[10] US Food and Drug Administration. ACAM2000 (smallpox vaccine) questions and answers, 2022. https://www.fda.gov/vaccines-blood-biologics/vaccines/acam2000-smallpox-vaccine-questions-and-answers

[11] World Health Organization. Overview of Mpox (monkeypox) vaccines and safety surveillance, 2023. https://www.who.int/groups/global-advisory-committee-on-vaccine-safety/topics/mpox

[12] Kenner J , Cameron F, Empig C et al. LC16m8: an attenuated smallpox vaccine. Vaccine 2006;24(47-48):7009–22. 10.1016/j.vaccine.2006.03.087 17052815 PMC7115618

[13] Saijo M , Ami Y, Suzaki Y et al. LC18m8, a highly attenuated vaccinia virus vaccine lacking expression of the membrane protein B5R, protects monkeys from monkeypox. J Virol 2006;80(11):5179–88. 10.1128/JVI.02642-05 16698998 PMC1472157

[14] Iizuka I , Ami Y, Suzaki Y et al. A single vaccination of nonhuman primates with highly attenuated smallpox vaccine, LC18m8, provides long-term protection against monkeypox. Jpn J Infect Dis 2017;70(4):408–15. 10.7883/yoken.JJID.2016.417 28003603

[15] Moriyama Y , Ujiie M. Monkeypox. N Engl J Med 2023;388(7):671. 10.1056/NEJMc2215751 36791179

[16] Wang L , Shang J, Weng S et al. Genomic annotation and molecular evolution of monkeypox virus outbreak in 2022. J Med Microbiol 2022;95(1):e28036. 10.1002/jmv.28036 PMC10087776 35906185

[17] Gao F , He C, Liu M et al. Cross-reactive immune responses to monkeypox virus induced by MVA vaccination in mice. Virol J 2023;20(1):126. 10.1186/s12985-023-02085-0 37337226 PMC10278293

[18] Frey SE , Wald A, Edupuganti S et al. Comparison of lyophilized versus liquid modified vaccinia Znkara(MVA) formulations and subcutaneous versus intradermal routes of administration in healthy vaccinia-naïve subjects. Vaccine 2025;33(39):5225–34. 10.1016/j.vaccine.2015.06.075 PMC9533873 26143613

[19] UK Health Security Agency. Monkeypox Vaccines to be Piloted in Smaller but Equally Effective Doses. London, UK: UK Health Security Agency press office, 2022. https://www.gov.uk/government/news/monkeypox-vaccines-to-be-piloted-in-smaller-but-equally-effective-doses

[20] Zaeck LM , Lamers MM, Verstrepen BE et al. Low levels of monkeypox virus-neutralizing antibodies after MVA-BN vaccination in healthy individuals. Nat Med 2023;29(1):270–8. 10.1038/s41591-022-02090-w 36257333 PMC9873555

[21] Sagy YW, Zucker R, Hammerman A et al. Real-world effectiveness of a single dose of mpox vaccine in males. Nat Med 2023;29(3):748–52. 10.1038/s41591-023-02229-3 36720271 PMC9930701

[22] Bertran M , Andrews N, Davison C et al. Effectiveness of one dose of MVA–BN smallpox vaccine against mpox in England using the case-coverage method: an observational study. Lancet Infect Dis 2023;23(7):828–35. 10.1016/s1473-3099(23)00057-9 36924787

[23] Lipsitch M , Boyer CB. Alternative analysis of mpox vaccine effectiveness data from Israel. *medRxiv*, 2023. 10.1101/2023.02.22.2328624

[24] UK Health Security Agency. Monkeypox: case definitions, 2022. https://www.gov.uk/guidance/monkeypox-case-definitions (23 January 2023, date last accessed).

[25] Thy M , Peiffer-Smadja N, Mailhe M et al. Breakthrough infections after postexposure vaccination against Mpox. N Engl J Med 2022;387(26):2477–9. 10.1056/NEJMc2211944 36477495

[26] Payne AB , Ray LC, Cole MM et al. Reduced risk for Mpox after receipt of 1 or 2 doses of JYNNEOS vaccine compared with risk among unvaccinated persons – 43 US jurisdictions, July 31–October 1, 2022. MMWR Morb Mortal Wkly Rep 2022;71(49):1560–4. 10.15585/mmwr.mm7149a5 36480479 PMC9762900

[27] Payne AB , Ray LC, Kugeler KJ et al. Incidence of monkeypox among unvaccinated persons compared with persons receiving ≥1 JYNNEOS vaccine dose – 32 US jurisdictions, July 31–September 3, 2022. MMWR Morb Mortal Wkly Rep 2022;71(40):1278–82. 10.15585/mmwr.mm7140e3 PMC9541026 36201401

[28] Morales LM et al . Post-exposure vaccine effectiveness and contact management in the mpox outbreak, Madrid, Spain, May to August 2022. Euro Surveill 2023;28(24):883. 10.2807/1560-7917.ES.2023.28.24.2200883 PMC10318941 37318762

[29] Taub DD , Ershler WB, Janowski M et al. Immunity from smallpox vaccine persists for decades: a longitudinal study. Am J Med 2008;121(12):1058–64. 10.1016/j.amjmed.2008.08.019 19028201 PMC2610468

[30] Hammarlund E , Lewis MW, Hansen SG et al. Duration of antiviral immunity after smallpox vaccination. Nat Med 2003;9(9):1131–7. 10.1038/nm917 12925846

[31] Kwanchum K , Ampol S, Thongput A et al. Duration of neutralizing antibody persisting in Thai individuals after childhood vaccination against smallpox. Asian Pac J Allergy Immunol 2017;35(4):239–43. 10.12932/AP0857 28577520

[32] Hsieh S-M , Pan S-C, Chen S-Y et al. Age distribution for T cell reactivity to vaccinia virus in a healthy population. Clin Infect Dis 2004;38(1):86–9. 10.1086/380460 14679452

[33] Hotez PJ , Brindley PJ, Bethony JM et al. Helminth infections: the great neglected tropical diseases. J Clin Invest 2008;118(4):1311–21. 10.1172/JCI34261 18382743 PMC2276811

[34] Mabbott NA. The influence of parasite infections on host immunity to co-infection with other pathogens. Front Immunol 2018;9:2579. 10.3389/fimmu.2018.02579 30467504 PMC6237250

[35] Wait LF , Dobson AP, Graham AL. Do parasite infections interfere with immunisation? A review and meta-analysis. Vaccine 2020;38(35):5582–90. 10.1016/j.vaccine.2020.06.064 32616328

[36] Egwang TG , Owalla TJ, Kemigisha M. COVID-19 vaccine trials must include helminth-infected cohorts. Nat Immunol 2022;23(2):148. 10.1038/s41590-021-01116-8 35075281

[37] Riser AP, Hanley A, Cima A et. al . Epidemiologic and clinical features of Mpox-associated deaths - United States, May 10, 2022 - March 7, 2023. Morb Mortal Wkly Rep 2023;72(15):404–10. 10.15585/mmwr.mm7215a5 PMC10121256 37053126

[38] Liu L , Iketani S, Guo Y et al. Striking antibody evasion manifested by the Omicron variant of SARS-Cov-2. Nature 2021;602(7898):676–81. 10.1038/s41586-021-04388-0 35016198

[39] Chalkias S , Harper C, Vrbicky K et al. A bivalent Omicron-containing bosster vaccine against Covid-19. N Engl J Med 2022;387(14):1279–91. 10.1056/nejmoa2208343 36112399 PMC9511634

[40] Jamard S et al . Resurgence of symptomatic Mpox among vaccinated patients: first clues from a new onset cluster. Infect Dis Now 2023;53(4):104714. 10.1016/j.idnow.2023.104714 37120092 PMC10156087

[41] Rcheulishvili N , Mao J, Papukashvili D et al. Design, evaluation, and immune simulation of potentially universal multi-epitope mpox vaccine candidate: focus on DNA vaccine. Front Micrbiol 2023;14:1203355. 10.3389/fmicb.2023.1203355 PMC10403236 37547674

[42] Tang D , Liu X, Lu J et al. Recombinant proteins A29L, M1R, A35R, and B6R vaccination protects mice from mpox virus challenge. Front Immunol 2023;14:1203410. 10.3389/fimmu.2023.1203410 37435062 PMC10331816

[43] Heraud J-M , Edghill-Smith Y, Ayala V et al. Subunit recombinant vaccine protects against monkeypox. J. Immunol 2006;177(4):2552–64. 10.4049/jimmunol.177.4.2552 16888017

[44] UK Health Security Agency. Mpox (monkeypox) outbreak: epidemiological overview, 2023. 25 May 2023 (interim update). https://www.gov.uk/government/publications/monkeypox-outbreak-epidemiological-overview/mpox-monkeypox-outbreak-epidemiological-overview-25-may-2023-interim-update

[45] US Centers for Disease Control and Prevention. Mpox Case Trends Reported to CDC, 2023. https://www.cdc.gov/poxvirus/mpox/response/2022/mpx-trends.html (31 May 2023, date last accessed).

[46] World Health Organization. Statement on the fifteenth meeting of the IHR (2005) Emergency Committee on the COVID-19 pandemic, 2023. https://www.who.int/news/item/05-05-2023-statement-on-the-fifteenth-meeting-of-the-international-health-regulations-(2005)-emergency-committee-regarding-the-coronavirus-disease-(covid-19)-pandemic

[47] UK Health Security Agency. People still eligible for Mpox vaccine urged to come forward, 2023. https://www.gov.uk/government/news/people-still-eligible-for-mpox-vaccine-urged-to-come-forward

[48] Nguyen PY , Ajisegiri WS, Costantino V et al. Reemergence of human monkeypox and declining population immunity in the context of urbanization, Nigeria, 2017–2020. Emerg Infect Dis 2021;27(4):1007–14. 10.3201/eid2704.203569 33756100 PMC8007331

